# Lymph node dissection before initial treatment for locally advanced cervical cancer: A systematic review and meta-analysis

**DOI:** 10.17305/bb.2024.10591

**Published:** 2024-12-01

**Authors:** He Zhang, Miao Ao, You Wu, Wei Mao, Haixia Luo, Kunyu Wang, Bin Li

**Affiliations:** 1 Department of Gynecological Oncology, National Cancer Center/National Clinical Research Center for Cancer/Cancer Hospital, Chinese Academy of Medical Sciences and Peking Union Medical College, Beijing, China

**Keywords:** Lymph node dissection, locally advanced cervical cancer, meta-analysis

## Abstract

The effectiveness of removing lymph nodes before initial treatment in patients with locally advanced cervical cancer is still debated. This article presents a meta-analysis that systematically evaluates the impact of this approach on oncological outcomes. A systematic literature search of PubMed, Embase, Science Direct, and the Cochrane Database of Systematic Reviews (up to December 2023) was performed to obtain relevant studies. The findings were combined using fixed-effects models to address potential differences. Combined risk ratios (HR) and 95% confidence intervals (CIs) were calculated. Egger’s test was used to assess publication bias. Out of 1,025 screened articles, four studies (involving 838 women) met the inclusion criteria. The results showed that lymph node dissection before initial treatment did not affect overall survival (OS) in patients with locally advanced cervical cancer compared to concurrent radiotherapy (HR ═ 1.11, 95% CI ═ 0.91–1.36, *P* ═ 0.30). It also did not increase the incidence of postoperative complications or cause delays in radiotherapy. In particular, removing larger lymph nodes (>2 cm) aided in defining the radiation field and decreasing radiotherapy-related complications. The surgical technique also had some impact on postoperative complications. In summary, in order to obtain the best therapeutic outcomes, personalized plans should be developed for each patient, accounting for their individual circumstances to achieve precise treatment and enhance their quality of life.

## Introduction

Cervical cancer is one of the most common malignant tumors of the female reproductive system and has a severe impact on women’s health. Based on estimates, China is projected to experience approximately 111,820 new cases and 61,579 deaths from this disease in 2022 [1]. Fortunately, early detection through screenings and the availability of the human papillomavirus vaccine have led to a decline in the incidence of cervical cancer. This results in a better prognosis for most patients who are typically diagnosed in the earlier stages [2]. Nevertheless, there are still some cases of advanced or locally advanced disease, often due to inadequate screening awareness. Locally advanced cervical cancer, according to the International Federation of Gynecology and Obstetrics (FIGO) definition, refers to cases classified as FIGO stage IIB to IVA [3]. Patients with this type of cervical cancer have a higher probability of lymph node metastasis, paracervical involvement, and lymphovascular infiltration, all of which are intermediate- and high-risk factors for recurrence. Their 5-year overall survival (OS) rate is also significantly lower, with reported rates as low as 50% to 60% [4].

Among them, lymph node metastasis is of great significance in the selection of treatment options for cervical cancer and patient prognosis [5]. The update to FIGO 2018 staging further validates this perspective [3]. The method of diagnosis of lymph node metastasis should be indicated along with the staging, with a note (r) for those diagnosed by imaging and a note (p) for those diagnosed by surgical staging. Although positron emission tomography/computed tomography (PET/CT) has replaced conventional CT and MRI as the gold standard for evaluating lymph node metastasis with the advancement of imaging technology, the false-negative rate of PET-CT for para-aortic lymph nodes (PALN) is still as high as 6%–15% [6]. According to the latest National Comprehensive Cancer Network (NCCN) guidelines, simultaneous radiotherapy is the primary means recommended by the guidelines for the treatment of locally advanced cervical cancer, in which radiotherapy is mainly pelvic field irradiation [7]. Patients with combined para-abdominal aortic lymph node metastasis are supplemented with expanded field irradiation [8–10]. However, in cases where imaging or surgical staging detects enlarged lymph nodes, radiotherapy may not be sufficient to eradicate them. Studies have shown that surgical resection or direct lymph node dissection can improve survival in these cases [11]. However, current guidelines remain controversial regarding the treatment options for enlarged lymph nodes in patients with locally advanced cervical cancer. In particular, there is controversy regarding the indications for surgery and whether surgery improves prognosis [12]. In addition, for enlarged nodes, the standard dose of conventional external irradiation (50–60 Gray) may not be sufficient for curative treatment, and additional treatment may be required [13–15]. Therefore, in locally advanced cervical cancer, assessment of lymph node metastasis prior to simultaneous radiotherapy is significant and helps to develop a more precise treatment plan [16].

For this reason, we designed this meta-analysis. The purpose of this meta-analysis was to investigate the impact of pre-treatment lymph node dissection on postoperative complications and patient survival in locally advanced cervical cancer. Our analysis is based on the existing literature and data with the aim of assessing the surgical management of this type of cancer.

## Materials and methods

### Study protocol

We conducted a systematic literature review and meta-analysis in accordance with the Cochrane Evaluation Methods Guidelines and the Preferred Reporting Items for Systematic Reviews and Meta-Analyses (PRISMA) guidelines [17]. Two independent investigators (HZ, MA) screened titles and abstracts against selected inclusion criteria. A third reviewer (YW) was asked to resolve any disagreements. This systematic review and meta-analysis have been registered in the International Prospective Register of Systematic Reviews (PROSPERO) with the number CRD42024492509.

### Search strategy

The principle of PICO, which is explained below, was utilized to determine the inclusion criteria for the meta-analysis. P (participant): patients with FIGO 2009 stage IB2, IIA2–IVA locally advanced cervical cancer of any age and histology. I (intervention): received lymph node dissection as initial treatment. C (control): received radiotherapy or chemotherapy only. O (outcome): patient’s survival index.

Our data were searched through the following databases: PubMed, Embase, Science Direct, and Cochrane Database of Systematic Reviews. Relevant reports and studies retrieved on ClinicalTrials.gov were also screened to identify relevant literature. The main search terms were cervical tumor, lymph node dissection, radiotherapy or chemotherapy, and survival with a December 2023 deadline. Surgical methods mainly included open, laparoscopic, or robotic surgery. The bibliographies of included articles were also thoroughly assessed and analyzed to locate additional studies. We excluded case reports or abstracts, video articles, review articles, review articles that did not report raw data, unpublished data, and duplicate publications. We also excluded ongoing studies as well as protocols. The search included only English-language articles. The overall search strategy is described in Table S1.

### Data extraction

The following data were extracted: authors, year of publication, country/region of study, number of patients, the median age of patients, body mass index (BMI), study period, surgical pathway, tumor stage, histological type, region of bulky node, adjuvant therapy, number of progression or recurrence, number of deaths, median follow-up date, OS, and postoperative complications. OS is the time from the date of diagnosis to death or last follow-up.

### Quality assessment

The risk of bias in the included cohort studies was assessed using the Newcastle–Ottawa Scale [18, 19]. The scale uses a star scoring system (up to 9 stars) to assess studies in terms of participant selection, comparability of study groups, and outcome ascertainment. Studies scoring 7 or above were classified as having a low risk of bias, those scoring between 5 and 6 stars as moderate risk of bias, and those with a score of 4 or less as high risk of bias.

### Publication bias

Egger’s test was used to assess publication bias. If the data points formed a symmetrical funnel-shaped distribution with a one-tailed significance level of *P* > 0.05 (Egger’s test), it indicated that there was no publication bias.

### Statistical analysis

We evaluated the overall disease survival difference between the lymphodepleted and non-lymphodepleted groups by using the extracted hazard ratio (HR) from time-to-event survival analysis. We extracted the HR values and their corresponding 95% confidence intervals (CIs) directly from the original articles. In the absence of this information, we calculated or extrapolated the relevant results using the Parmar et al. [20] and Williamson et al. [21] methods based on the provided Kaplan–Meier curves.

To determine the appropriate statistical model, meta-analyses were conducted based on heterogeneity between studies. The assessment of heterogeneity relied on two statistics: the chi-square test based on Cochran’s *q*-test and the *i*-squared statistic. If the *i*-squared statistic showed significant heterogeneity (>50%), we used a random-effects model, treating these studies as random samples from a hypothetical population with different effects [22]. In all cases, study weights were determined using an inverse variance approach. A two-sided *P* value of less than 0.05 was considered statistically significant when calculating combined effects. The R-4.0.4 software was used for statistical analyses and visualization.

**Table 1:** Basic characteristics of included studies in the meta-analysis

**Author**	**Year**	**Study period**	**Patients (*n*)**	**Average** **age (years)**	**BMI** **(kg/m^2^)**	**Country/ Region**	**Enlarged pelvic nodes**	**FIGO stage (*n*)**				**Histologic type (*n*)**		**Study group (*n*)**	**Control group (*n*)**	**Progression and recurrence (*n*)**	**Death (*n*)**	**Median follow-up (months)**
								**IB2**	**II**	**III**	**IVA**	**Squamous**	**Non-squamous**					
Chen	2012	1993–2001	56	73	NA	Taiwan	NA	NA	24	31	1	NA	NA	19	37	22	34	NA
Marnitz	2020	2009–2013	240	48.4	26.2	German	NA	NA	165	63	12	211	29	121	119	95	102	NA
Díaz-Feijoo	2022	2000–2016	381	49	25.9	Spain	>1 cm	64	222	82	13	308	73	275	106	123	148	44.4
Olthof	2022	2009–2017	161	51	NA	The Netherlands	>1.5 cm	29	87	39	6	140	21	101	60	80	NA	46

FIGO: International Federation of Gynecology and Obstetrics; NA: Not applicable; IB2: Cancer clinically visible; IVA: Cancer spread beyond the true pelvis.

## Results

### Search results

**Figure 1.**
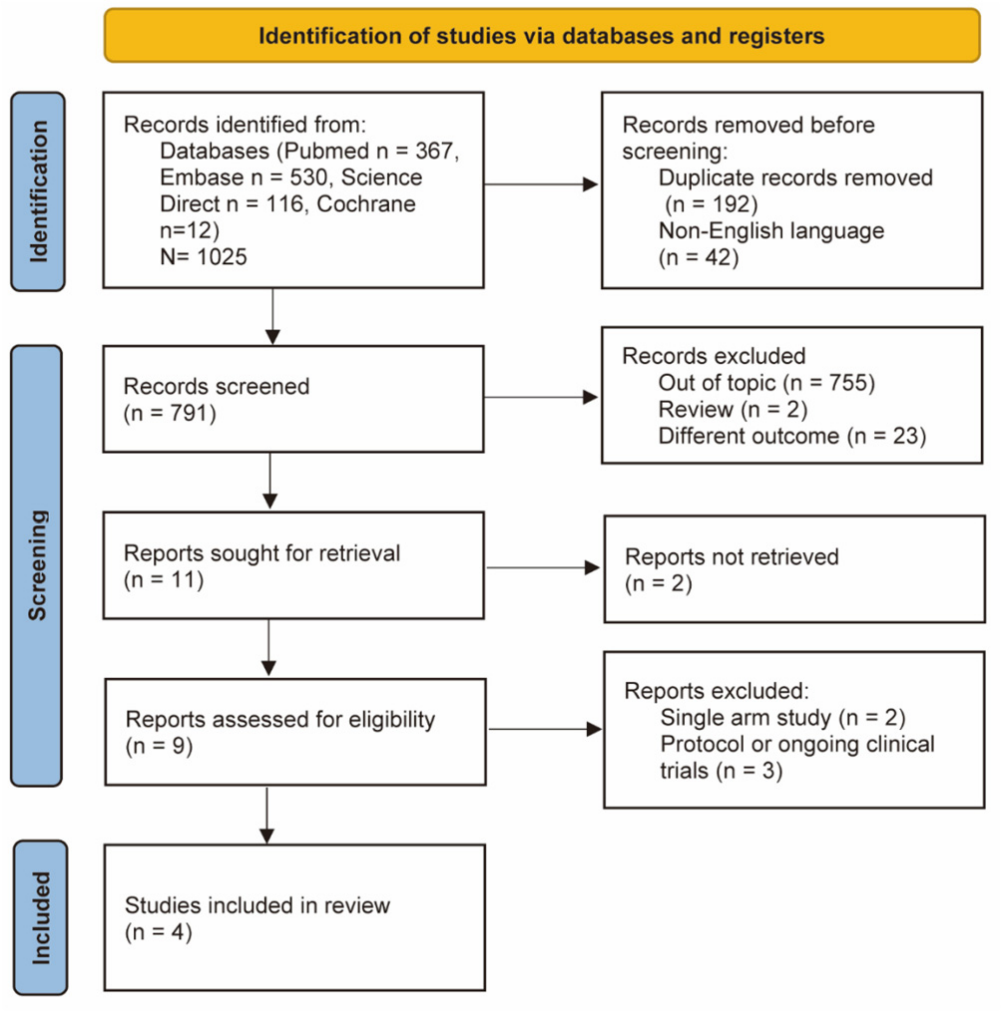
**PRISMA flow diagram for study identification and inclusion**. PRISMA: Preferred Reporting Items for Systematic Reviews and Meta-Analyses.

Figure 1 gives a flowchart of the research retrieval and selection process for this paper. After eliminating duplicates and non-English literature, our initial literature search yielded 791 articles reviewed for titles and abstracts. We excluded 755 studies that were not relevant to the review topic. Of the nine articles selected for full-text review, two were single-arm studies [23, 24], and three were ongoing clinical trials or protocols [25–27], resulting in a total of four studies that met all inclusion criteria [28–31]. Table 1 provides more details of the included studies.

This scientific article included four articles in the systematic review and meta-analysis after screening. Of these, one was a prospective randomized international multi-center-controlled study [30] and three were retrospective observational studies [28, 29, 31]. Published between 2012 and 2022, these studies were conducted in Spain, The Netherlands, Germany, and Taiwan. A total of 838 patients, with a mean age of 50.8 years, were included in the studies. The sample size varied from 19 to 275 in the lymphatic clearance group and 37 to 106 in the non-lymphatic clearance group. The number and site of lymph node dissection and surgical access varied across studies, including transabdominal, laparoscopic, and robotic lymph node dissection. Only two articles addressed different aspects of postoperative complications and toxic reactions and therefore were not included in this meta-analysis [29, 31]. All four studies provided patient survival information, including OS data. The study by Chen et al. also reported failure-free survival (FFS) data, while Díaz-Feijoo and Olthof included disease-free survival (DFS) and relapse-free survival (RFS) data, respectively. Multivariate analyses had varying adjustment factors and follow-up durations.

### Quality assessment

We assessed the quality of the included cohort studies using the Newcastle–Ottawa scale. All four studies achieved a score of seven or higher, indicating a low risk of bias. For more details on the risk of bias assessment, please refer to Table S2.

### Meta-analysis for OS

**Figure 2.**
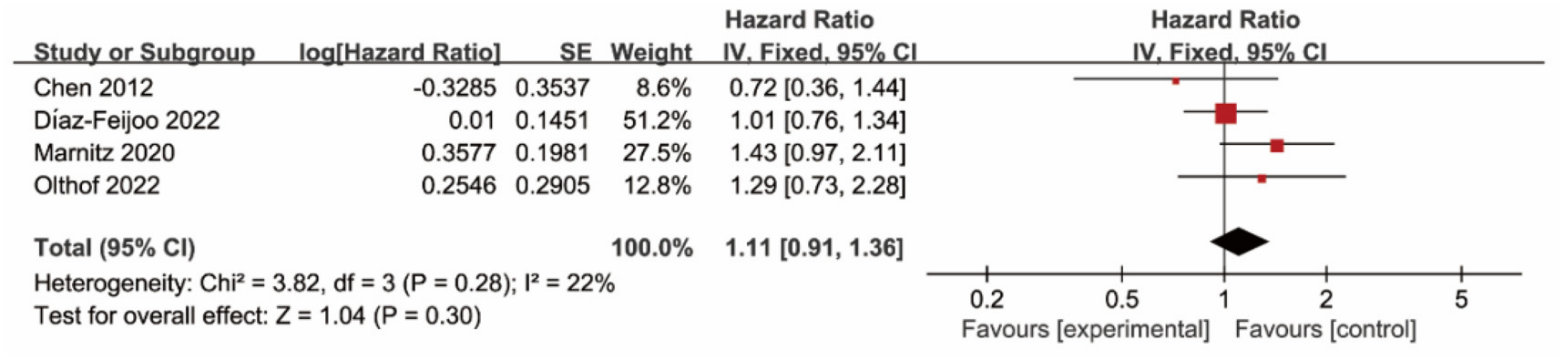
**Forest plot of OS in patients with locally advanced cervical cancer with and without lymph node dissection**. OS: Overall survival; CI: confidence interval.

Our study included 936 female patients from four studies and utilized a fixed-effects model (rank-sum ═ 3.82; *I*^2^ ═ 22%; *P* ═ 0.28) to analyze the data. Our findings indicate that pre-treatment lymph node dissection does not significantly impact OS in patients with locally advanced cervical cancer (HR ═ 1.11; 95% CI ═ 0.91–1.36; *P* ═ 0.30). Figure 2 displays the results of our meta-analysis and the forest plot.

### Publication bias

**Figure 3.**
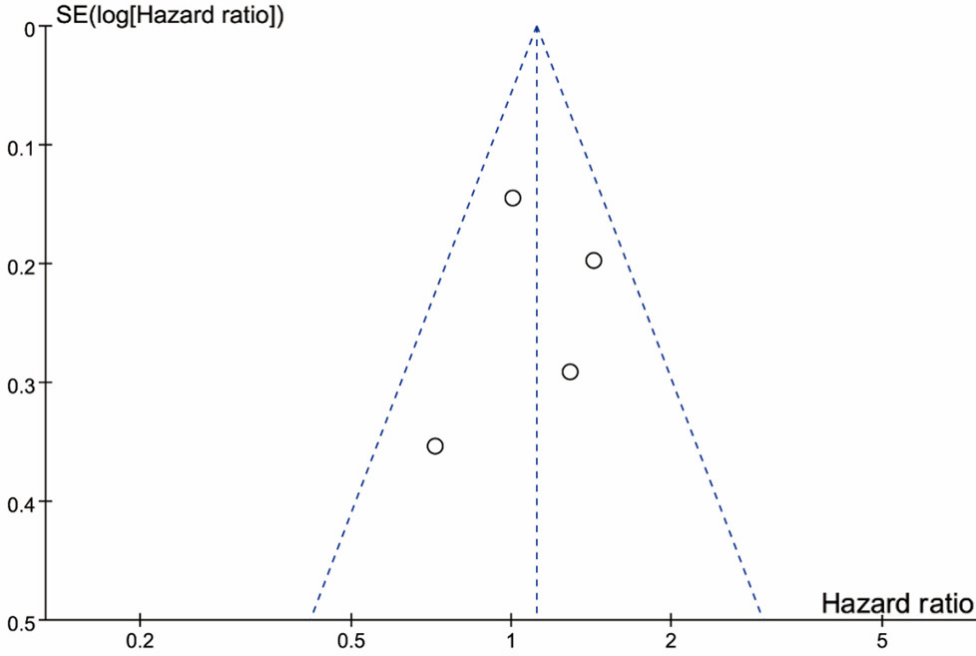
**Funnel plots of standard error by hazard ratio of OS for patients with and without lymphadenectomy**. OS: Overall survival; SE: Standard error.

There was no evidence of significant publication bias by inspection of the formal statistical tests (Egger’s test). A detailed publication bias assessment is described in Figure 3.

## Discussion

This study is the first systematic review and meta-analysis to investigate the necessity of removing lymph nodes before initial treatment for locally advanced cervical cancer. The findings of the meta-analysis showed no significant difference in OS between patients who underwent lymph node dissection and those who received simultaneous radiotherapy. Several similar studies have shown comparable 5-year recurrence-free survival rates for patients with microscopic and macroscopic lymph node metastases resected prior to initial treatment (50%–57% and 43%–57%, respectively), compared with a 0% survival rate for patients with unresectable metastatic lymph nodes [23, 24]. All of the above studies came to similar conclusions, i.e., they illustrated that removing metastatic enlarged lymph nodes did not affect patient survival. Díaz-Feijoo’s study also showed that the difference in recurrence rate after treatment was not statistically significant in the lymph node dissection group compared to the non-lymph node dissection group [32].

For surgical access, the conclusions of the Uterus-11 study suggest that removal of lymph nodes by laparoscopic surgery avoids serious complications during subsequent radiation therapy [11]. The complication rates for laparoscopic surgery without delaying subsequent radiation therapy ranged from 1.6% to 7%, compared with a 34% complication rate for open surgery with subsequent radiation therapy [32–35]. However, there is controversy regarding the extent of para-aortic lymph node dissection at the level of the renal vessels or the level of the inferior mesenteric artery for a variety of laparoscopic surgical approaches and modalities, including transperitoneal or retroperitoneal approach, conventional laparoscopic or robotic laparoscopic surgery. Further prospective randomized controlled trials are expected to be published [33, 35–37].

Additionally, the removal of enlarged positive lymph nodes may improve survival. This is because large lymph nodes are more difficult to completely eradicate with radiotherapy and may not be included in the radiation field [37, 38]. Wakatsuki et al.’s study found that the control rate for cervical cancer patients treated with 50-Gy radiotherapy was 97% for lymph nodes smaller than 10 mm and 76% for lymph nodes larger than 10 mm. The field failure rate of pelvic and paraaortic lymph nodes >10 mm was significantly higher than that of smaller lymph nodes. Oh et al. found similar results in an 83-month follow-up of 310 patients with locally advanced cervical cancer.

Olthof’s study performed a subgroup analysis of enlarged lymph nodes ≥2 cm. However, the two groups had no significant difference between 5-year OS (*P* ═ 0.83) and RFS (*P* ═ 0.91). In multivariate analysis, different treatment strategies did not affect OS and RFS. There was also no difference in toxicity [31]. These results may be related to the small number of patients enrolled. Therefore, the removal of larger lymph nodes may be considered in order to enhance the local control rate with radiation therapy. In addition, lymph node dissection before initial treatment can be used to conduct pathological evaluation of lymph node tissue and determine surgical staging [39, 40]. It has been reported that surgical removal of lymph nodes can improve the therapeutic effect by about 20%–40% compared to PET-CT results [41, 42]. Surgical removal of the lymph nodes can also accurately map out the radiation field and reduce radiation complications.

Recent studies have shown that lymph node dissection before initial treatment enables pathologic evaluation of lymph node tissue, validates imaging findings, and improves diagnostic accuracy. Surgical removal of lymph nodes can result in approximately 20%–40% improvement in treatment compared to PET-CT findings. In addition, the removal of enlarged positive lymph nodes may provide therapeutic benefits. This may be related to the difficulty of eradicating large lymph nodes with radiotherapy and the fact that it ensured that the lymph nodes diagnosed were included in the radiation field.

This is the first review and analysis to examine the necessity of lymph node dissection prior to initial treatment for locally advanced cervical cancer, and it will be useful for clinicians to implement clinical decisions. This study still has some shortcomings and flaws. First, the included clinical studies were retrospective, which may impact the results. Second, the number of studies analyzed was small, potentially impacting the validity of the findings. According to the retrieved literature, two new randomized controlled trials (Casper, NTR4922), (He, NCT04555226) have been initiated [26, 27].

In summary, surgery before initial treatment of locally advanced cervical cancer maximizes the removal of lymph nodes, significantly enlarged lymph nodes, and does not affect the occurrence of postoperative complications or the prognosis and survival of patients [43]. Postoperative simultaneous radiotherapy also does not cause delays due to prior surgery. Defining lymph node pathology and surgical staging will also lead to more precise postoperative radiotherapy fields, allowing individualized radiotherapy for patients with locally advanced cervical cancer, thus reducing or eliminating overtreatment of patients due to false-positive imaging and reducing radiotherapy-related complications.

## Conclusion

In conclusion, in patients with locally advanced cervical cancer, removal of lymph nodes before initial treatment does not provide a clear survival benefit. However, it can aid in identifying the extent of metastasis and does not increase surgical complications. This allows for precise determination of the radiotherapy area and avoids unnecessary treatment complications for patients without lymph node involvement. Gynecologic oncologists should consider tailored treatment strategies for patients with locally advanced cervical cancer in high-risk groups, especially those at risk for lymph node metastasis. Additionally, efforts should be made to accurately assess lymphatic involvement before initial treatment in order to identify those who would benefit from lymphatic cleansing. To validate the impact of pelvic lymph node dissection in this population, further randomized controlled studies are necessary.

## Supplemental data

**Table S1:** Search strategy

**No.**	**Search query**	**PubMed**
#1	(Uterine Cervical Neoplasms [Mesh]) OR (Cervical Neoplasm, Uterine) OR (Neoplasm, Uterine Cervical) OR (Uterine Cervical Neoplasm) OR (Neoplasms, Cervical) OR (Cervical Neoplasms) OR (Cervical Neoplasm) OR (Neoplasms, Cervix) OR (Cervix Neoplasm) OR (Neoplasm, Cervix) OR (Cervix Neoplasms) OR (Cancer of the Uterine Cervix) OR (Cancer of the Cervix) OR (Cervical Cancer) OR (Cancer, Cervical) OR (Cervical Cancers) OR (Uterine Cervical Cancer) OR (Cancer, Uterine Cervical) OR (Cervical Cancer, Uterine) OR (Uterine Cervical Cancers) OR (Cancer of Cervix) OR (Cervix Cancer) OR (Cancer, Cervix)	152070
#2	(Lymph Node Excision [Mesh]) OR (Excision, Lymph Node) OR (Excisions, Lymph Node) OR (Lymph Node Excisions) OR (Lymphadenectomy) OR (Lymphadenectomies) OR (Lymph Node Dissection) OR (Dissection, Lymph Node) OR (Dissections, Lymph Node) OR (Lymph Node Dissections) OR (Node Dissection, Lymph) OR (Node Dissections, Lymph)	82000
#3	(Radiotherapy [Mesh]) OR (Radiotherapies) OR (Radiation Therapy) OR (Radiation Therapies) OR (Therapies, Radiation) OR (Therapy, Radiation) OR (Radiation Treatment) OR (Radiation Treatments) OR (Treatment, Radiation) OR (Radiotherapy, Targeted) OR (Radiotherapies, Targeted) OR (Targeted Radiotherapies) OR (Targeted Radiotherapy) OR (Targeted Radiation Therapy) OR (Radiation Therapies, Targeted) OR (Targeted Radiation Therapies) OR (Therapies, Targeted Radiation) OR (Therapy, Targeted Radiation) OR (Chemoradiotherapy [Mesh]) OR (Radiation Therapy, Targeted) OR (Chemoradiotherapies) OR (Radiochemotherapy) OR (Radiochemotherapies) OR (Concurrent Chemoradiotherapy) OR (Chemoradiotherapies, Concurrent) OR (Chemoradiotherapy, Concurrent) OR (Concurrent Chemoradiotherapies) OR (Synchronous Chemoradiotherapy) OR (Chemoradiotherapies, Synchronous) OR (Chemoradiotherapy, Synchronous) OR (Synchronous Chemoradiotherapies) OR (Concurrent Radiochemotherapy) OR (Concurrent Radiochemotherapies) OR (Radiochemotherapies, Concurrent) OR (Radiochemotherapy, Concurrent) OR (Concomitant Chemoradiotherapy) OR (Chemoradiotherapies, Concomitant) OR (Chemoradiotherapy, Concomitant) OR (Concomitant Chemoradiotherapies) OR (Concomitant Radiochemotherapy) OR (Concomitant Radiochemotherapies) OR (Radiochemotherapies, Concomitant) OR (Radiochemotherapy, Concomitant)	607487
#4	((randomized controlled trial[pt] OR (controlled clinical trial[pt]) OR (randomized[tiab]) OR (randomised[tiab]) OR (placebo[tiab]) OR (randomly[tiab]) OR (trial[tiab]) OR (groups[tiab])) NOT (animals[mh] NOT humans[mh]))	3345134
#5	(Survival) OR (Disease-Free Survival) OR (Progression-Free Survival) OR (Prognosis [Mesh]) OR (Prognoses) OR (Prognostic Factors) OR (Prognostic Factor) OR (Factor, Prognostic) OR (Factors, Prognostic)	4176384
#6	#1 AND #2 AND #3 AND #4 AND #5	367

pt: Publication type; tiab: Title/abstract; mh: Medical subject headings.

**Table S2:** Risk of bias assessment of the included cohort studies

**Study, year**	**Selection**				**Comparability**	**Outcome**			**Total score**
	**Exposed cohort**	**Non-exposed cohort**	**Ascertainment of exposure**	**Outcome of interest**		**Assessment of outcome**	**Length of follow-up**	**Adequacy of follow-up**	
Chen et al., 2012	☆	☆	☆	–	☆	☆	☆	☆	7
Marnitz et al., 2020	☆	☆	☆	☆	☆☆	☆	☆	☆	9
Díaz-Feijoo et al., 2022	☆	☆	☆	–	☆	☆	☆	☆	7
Olthof et al., 2022	☆	☆	☆	–	☆☆	☆	☆	☆	8

Risk of bias was evaluated with use of the Newcastle–Ottawa Scale. A score of 7 or higher indicates a low risk of bias.

## Data Availability

All data relevant to the study are included in the article or uploaded as supplementary information.
